# Design of Particulate-Reinforced Composite Materials

**DOI:** 10.3390/ma11020234

**Published:** 2018-02-03

**Authors:** Aleksander Muc, Marek Barski

**Affiliations:** Institute of Machine Design, Cracow University of Technology, 31-864 Kraków, Poland; mbar@mech.pk.edu.pl

**Keywords:** particulate-reinforced composite materials, homogenization, effective field method, numerical analysis, optimal design

## Abstract

A microstructure-based model is developed to study the effective anisotropic properties (magnetic, dielectric or thermal) of two-phase particle-filled composites. The Green’s function technique and the effective field method are used to theoretically derive the homogenized (averaged) properties for a representative volume element containing isolated inclusion and infinite, chain-structured particles. Those results are compared with the finite element approximations conducted for the assumed representative volume element. In addition, the Maxwell–Garnett model is retrieved as a special case when particle interactions are not considered. We also give some information on the optimal design of the effective anisotropic properties taking into account the shape of magnetic particles.

## 1. Introduction

Temperature as well as magnetic and electric fields in composite materials are of interest in many engineering applications. To use them effectively in modern constructions, it is necessary to predict the effective homogenized properties. Theoretically, for particle-filled, two-phase composites, their homogenized, effective physical properties may be derived in manner similar to the linear case. The methods for searching for effective magnetic permeabilities, dielectric permittivities or thermal conductivities are analogous and they may be calculated using the same approach. Although the similarity of those three fields was noticed a long time ago, the models for the prediction of the effective composite properties are built separately for each class of problems and in addition with the use of various simplified hypothesis (assumptions).

It is worth pointing out that the analysis of an incompressible viscous fluid flow through a porous medium can be described using analogous equations such as the ones mentioned above for the two-phase composites. On the macroscopic scale, flow through the porous material is governed by Darcy’s law having the permeability tensor K_H_, so that the methods, which are analogous to the ones discussed herein may also be successfully applied to the fluid flow problems.

There are different homogenization approaches and they can be divided into three classes, i.e., direct, indirect and variational methods. Direct methods are based on the volume average of field quantities and they can be performed by a numerical procedure, usually the finite element method (FEM) or the boundary element method (BEM). Indirect homogenization follows the idea of the equivalent inclusion method based on Eshelby’s eigenstrain solution. In this area, different variants of solutions are developed and they may be divided into: the self-consistent schemes, the Mori–Tanaka method and the differential method. The variational approach can give upper and lower bounds of the effective properties. Monographs describing different homogenization methods in details have been written by Mura [1], Nemat-Nasser and Hori [2], and Qin and Yang [3]. The review presented by Wang and Pan [4] first examines the issues, difficulties and challenges in the prediction of material behaviors by summarizing and critiquing the existing major analytical approaches dealing with material property modeling.

The model of Maxwell and Garnett [5,6] was one of the first to describe the effective permittivity of composites containing randomly dispersed spherical particles. Hashin and Shtrikman [7] used a variational approach to determine the upper and lower bounds of the effective magnetic permeability of multiphase materials. To investigate the effect of the interaction between particles, Fu et al. [8] presented an analytic approach to derive the explicit effective permittivity in the form of a series for composites containing spherical particles. For periodically distributed composites, McPhedran and McKenzie [9] as well as McKenzie et al. [10] extended a method devised by Rayleigh [11] to calculate the conductivity of simple cubic, body-centered cubic, and face-centered cubic lattices of composites containing conducting spheres. Doyle [12] and Lam [13] presented their models for composites with cubic lattices of particles. White [14] introduced a T-matrix solution based on a unit cell for general periodically distributed composites. Chen et al. [15] obtained the electric field distribution numerically in a Legendre series for one chain embedded in an infinite medium. Yin and Sun [16] derived effective magnetic properties for a chain-like structure considering a single column of particles embedded in an infinite medium. Sareni et al. [17,18] applied the boundary integral method to solve the effective permittivity for random and periodic composites. A broad review of the above-mentioned approaches and methods with the particular discussion of the results is given in the monograph of Kanaun and Levin [19]. In addition to the experimental and theoretical approaches, computer simulations have been adopted more and more frequently to study the effective properties of composites. Effective dielectric constants of two-phase composite dielectrics have been estimated numerically by Wu et al. [20]. Krakovsky and Myroshnychenko [21] used the finite element method to compute the effective permittivity for two-dimensional random composites. A numerical approach to the evaluation of the effective properties for magneto-rheological fluids (MR fluids) is demonstrated in reference [22], however, the analysis and results deal with the prediction of magnetic permeability in one direction only. Although materials with directional features are common, most previous work was focused on isotropic cases, except for some studies, which attempted to bring this property-direction dependence into the general formulation. For instance, for MR fluids, some magnetic properties, such as the saturation magnetization and the crystalline anisotropy, are intrinsic and depend mainly on the chemical composition and the crystalline symmetry of the material. On the other hand, extrinsic properties, such as remanence, coercivity and permeability, depend largely on the structure of the material (Yin and Sun [16]) or on the shape of reinforced particles (Vereda [23]). Through analytical predictions and numerical modeling, certain optimization approaches and design schemes for novel materials could be used for engineering applications and, in turn, the new observations and experiences from the practice would accelerate the development of new theories and methodologies.

By definition, smart materials have some properties which can be altered or tuned using an external field. Here, the works by Cai et al. [24], Moiseev et al. [25] or Moiseev [26] can be quoted. Other examples also include materials that exhibit ferroelectricity, pyroelectricity, piezoelectricity, a shape memory effect, electrostriction, magnetostriction electrochromism, photomagnetism and photochromism. Most of these materials tend to be used in their solid state, i.e., in a polycrystalline or a single crystal form as bulk materials or thin films deposited on appropriate substrates. In general, they form a special class of two-phase composite materials. When an external field is applied, the particles become polarized and are thereby arranged into chains or clusters. The chains can further aggregate into columns, when the composite material exhibits a solid-like mechanical behavior. Therefore, the effective properties of smart materials vary in time, starting from a random state of particles in a viscous fluid, and finishing in a composite solid as a chain-like structure.

To demonstrate the theoretical limitations, this study firstly aims to present the possibility of theoretical predictions of effective properties for smart materials in the 3D approach. The preliminary results are discussed in the papers by Barski and Muc [27] and Barski [28]. The presented theoretical approach is based on the use of the effective field method. Having in mind the possible applications of two-phase composites as smart materials, two separate problems are discussed in detail, i.e., the case of isolated inclusions and chain-like structures. Then, the numerical method of homogenization is proposed and the results are compared with the theoretical ones. The suggested scheme of numerical homogenization is applied to optimize the effective properties varying the shape of inclusions. As it is reported in the literature, the behavior of aggregated particles has a great influence on the appropriate modeling of the smart material deformation as it is strained and especially in view of its yield stress value. For such a class of composites, the present analysis is an introduction to the global FEM modeling of rheological deformations.

## 2. The Effective Field Method

**Figure 1:**
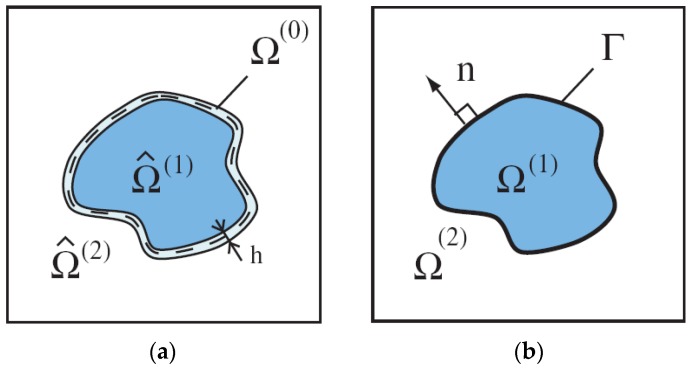
Fields in a homogeneous medium with inclusions: (**a**) particles with interfaces; and (**b**) two phase composites.

First, let us note that in engineering practice particles embedded in a matrix are usually coated by an additional material (an interface) between constituents (Figure 1) to enhance various properties of composites, i.e., thermal, magnetic or dielectric. Surfactants are added to alleviate the settling problem. Thus, a particulate composite is made of three different phases. Such a class of problems is analysed for instance by Kamiński [29]. Since it is very difficult to estimate the physical properties of an interface, our analysis is limited to the considerations of the two-phase material demonstrated in Figure 1b.

Variety of physical problems dealing with two-phase composites, understood in the sense of particles embedded in matrix, are described in the similar manner, i.e., with the use of similar physical equations. The examples of such relations are given in Table 1. For two-phased composites the general mathematical formulation of the problem as well as the methodology of the derivation of the effective material properties are presented in the Appendix A. The general symbols, which are used in the Appendix A, are explained in the first column in Table 1. However, the further numerical investigations concern the examples of the evaluation of effective magnetic permeability.

Below, two particular cases are solved to demonstrate the effectiveness and usefulness of the approach.

### 2.1. Isolated Inclusions

Let the matrix material be isotropic ($C_{\alpha \beta }^{0}=c_{0}\delta_{\alpha \beta }$) and the isotropic inclusions be ellipsoids with the same sizes randomly oriented in space. Neglecting the pair interaction between inclusions, the analysis is reduced to the consideration of a single inclusion embedded in an infinite medium. Thus, the effective properties of the two-phase composite can be easily computed from the relation in Equation (A21) (see the Appendix A) with the use of the definition in Equation (A11). For instance, in the case of spherical inclusions (so-called spherical symmetry), the operator $A_{\lambda \mu }^{0}$ takes the following form:

(1) $$A_{\lambda \mu }^{0}=\frac{1}{{3c}_{0}}\delta_{\lambda \mu }$$

the homogenized isotropic properties in Equation (A21) coincide with the well-known Maxwell–Garnett formula [5,6].

### 2.2. Chain of Inclusions

Using the above methodology, the effective properties of two-phase composites may also be found for materials with regular lattices of identical inclusions. In this case, the function $\Psi \left(x,x^{\prime }\right)$ in Equation (A16) depends on the difference x-x′. From that definition one can find that:

(2) $$\Psi \left(x\right)=\frac{\langle V(x^{\prime },x^{\prime }+x)|x^{\prime }\rangle }{\langle V(x)\rangle }=\frac{\langle V(x^{\prime },x^{\prime }+x)V(x^{\prime })\rangle }{{\langle V(x)\rangle }^{2}}$$

For the *i*-th inclusion being in the regular lattice, the integral

(3) $$\int V_{i}(x^{\prime })V_{i}(x^{\prime }+x){\mathrm{dx}}^{\prime }=\frac{4}{3}\pi a_{1}a_{2}a_{3}J\left(a\right),\;J\left(a\right)=\{\begin{array}{ll} (1-0.5{\left|x/a\right|}^{2})(1+\left|x/a\right|/4), & \left|x/a\right|\leq 2\\ 0, & \left|x/a\right|>2 \end{array}$$

is the volume of the intersection of two identical ellipsoids with the center separated by the vector x. Using the above definition, the numerator in Equation (2) can be written as follows:

(4) $$\langle V(x^{\prime },x^{\prime }+x)V(x^{\prime })\rangle =p\sum_{m}J(x-m)$$

where m is the vector of the lattice composed by the centers of inclusions. Thus, the function Ψ(x) can be expressed as follows:

(5) $$\Psi (x)=\frac{1}{p}\sum_{m}{}^{\prime }J(x-m)$$

where the prime over the sum symbol denotes the exclusion of the term with m = 0. Inserting the above results into the relation in Equation (A17), one can find that:

(6) $$K_{}^{\Psi }=\int K(x-x^{\prime })\Psi (x-x^{\prime }){\mathrm{dx}}^{\prime }=-A^{0}+\sum_{m}{}^{\prime }p.v.\int K(x)\left[\frac{1}{p}J(x-m)-1\right]\mathrm{dx}$$

and the symbol *p.v.* means that the integral is understood in the sense of the Cauchy principal value. Finally, with using Equations (A21) and (6), one can evaluate the effective properties of the composite with a regular lattice.

**Figure 2:**
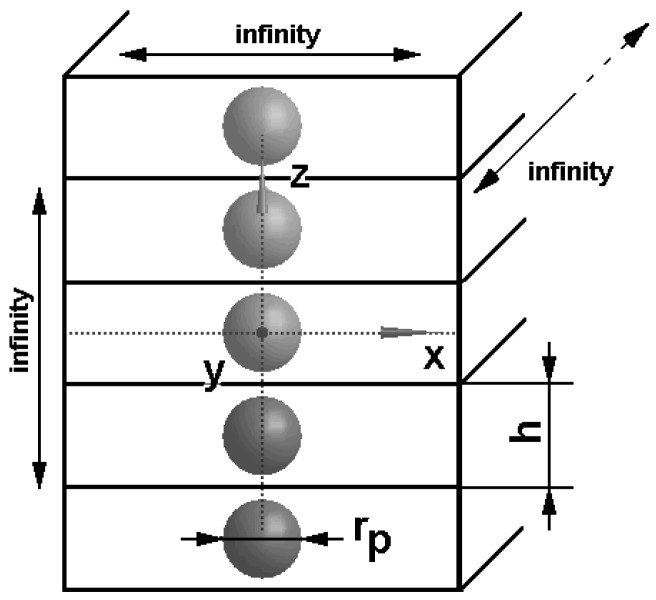
Chain of spherical inclusions.

It is worth emphasizing that the above definition of the effective mechanical properties does not consider the physical interaction between particles but the geometrical form of the assumed elementary cells only. The analytical results of integration in Equation (6) can be obtained for specific forms of regular lattices only. For instance, such a formula can be derived for an infinite chain of spherical particles where the representative volume cell has the form of a cuboid having an infinite length in two directions (Figure 2). In this case, the effective properties can be expressed in the following form:

(7) $$\begin{array}{l} C_{ii}^{*}=\left[C^{0}+pC_{1}{\left\{1+\left(1-p\right)C^{1}A^{0}+p\eta^{1}C^{1}\right\}}^{-1}\right],\;i=x,y\\ C_{zz}^{*}=\left[C^{0}+pC_{1}{\left\{1+\left(1-p\right)C^{1}A^{0}+p\eta^{3}C^{1}\right\}}^{-1}\right]\\ \eta^{1}=2\rho_{0}^{3}\sum_{m=1}^{\infty }1/m^{3},\;\eta^{3}=-2\eta^{1},\;\rho_{0}=r_{p}/h \end{array}$$

assuming the isotropic properties of the matrix and inclusions, and the operator A^0^ is described by Equation (1).

## 3. Numerical Homogenization Strategy

All known analytical methods are valid under certain limitations and particular geometries or classes of structures. For metamaterials comprising conducting and possibly resonant elements, and for which the periodicity is not necessarily negligible relative to the free-space wavelength, analytical homogenization techniques are unreliable or not applicable. Our intent here is to justify a numerically based homogenization scheme based on Table 1, in which the local fields computed for one unit cell of a periodic structure are averaged to yield a set of macroscopic fields. Having computed the macroscopic fields, we can then determine the constitutive relationships between the macroscopic fields, arriving at the effective electromagnetic parameters. We will see that there will be virtually no restrictions on the contents of the unit cell, nor will the unit cell necessarily need to be small in comparison with the wavelength. They have proven useful in many situations, e.g., low volume fraction of homogeneous spherical or ellipsoidal inclusions in a homogeneous host material, but they fail if the volume fraction is too high or if the inclusions are not spheres or ellipsoids. Then, a more accurate homogenization procedure has to be used, which includes all contributions of the interaction between the reinforcing particles.

**Figure 3:**
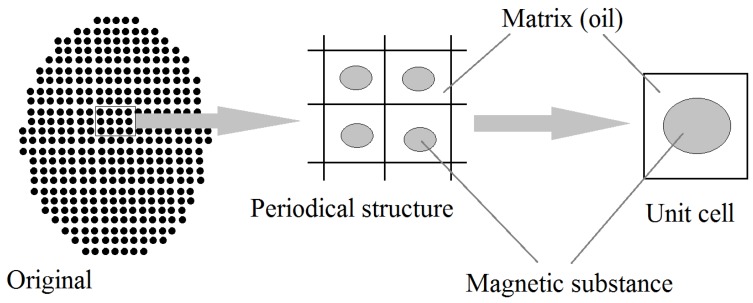
Definition of a unit cell.

For two-phase composites, a typical homogenization situation is depicted in Figure 3. It shows a 2D model of the composite material, which includes a matrix with inclusions. In the homogenization method, the structure of the two-phase composite materials is assumed to be periodic, and the unit cell, which is the minimum volume to represent the overall statistics, is defined. Here, it is assumed that one particle inclusion is located in the center of the cell. The unit cell is regarded as a homogeneous substance with the effective properties. The effective property is defined based on energy balance in the unit cell (Figure 3). It is assumed that the original cell and the homogenized cell include equivalent energy when both unit cells are immersed in equivalent external field.

In an actual estimation, the solution of the Laplace equation obtained with the use of basic relation shown in Table 1 is computed by FEM. In this analysis, the potential ϕ is unknown, and, assuming the applied field is unidirectional at least in the cell, the boundary conditions are set as:

(8) $$\varphi =0\;\mathrm{at}\;\mathrm{the}\;\mathrm{bottom}\;x=0,\;\varphi =E_{y}x\;\mathrm{at}\;\mathrm{the}\;\mathrm{top}\;x=b/2$$

**Figure 4:**
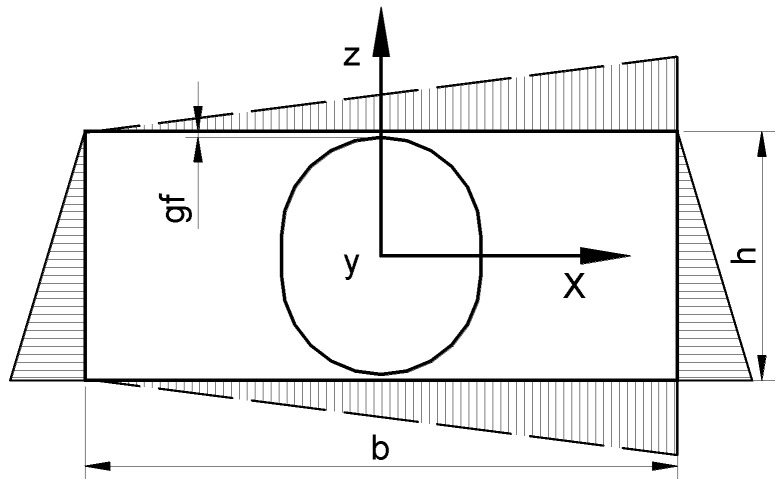
The geometry of the representative unit cell and of the boundary conditions—2D problem.

The identical boundary conditions are formulated at each parallel boundaries of the cell shown in Figure 4 for the 2D case. Let us note that the above type of boundary conditions satisfies the periodicity of boundary conditions for the arbitrary type of regular lattices as well as for the chain of inclusions and for a single inclusion.

Similar to the previous case for the effective field method, the FEM analysis is based on the averaging method that is carried out for the representative volume element (RVE) having the volume denoted by the symbol Ω_RVE_. Thus, it is obvious that the results, understood in the sense of the average property tensor, are directly dependent on the dimensions and form of the RVE. It is worth pointing out that, even for the 2D two-phase periodic composites, the RVE may be of an arbitrary form, not necessarily rectangular as it shown in Figure 3. Since in our numerical analysis we intend to give information on the variations of the property tensor components with respect to the volume fraction p, we define it in the following way (see Figure 4):

(9) $$p=\frac{\pi r_{p}^{2}}{2{\mathrm{br}}_{p}\left(1+g_{f}/r_{p}\right)}\;\mathrm{for}\;2D\;\mathrm{and}\;p=\frac{2\pi r_{p}^{3}}{3b^{2}r_{p}\left(1+g_{f}/r_{p}\right)}\;\mathrm{for}\;3D$$

where it is assumed that the RVE has a square cross-section in the y direction. Thus, for the prescribed volume fraction p, the RVE is completely defined by the set of two parameters (geometrical ratios), i.e., g_f_/r_p_ and b/r_p_ for both 2D and 3D cases. Let us note that, for the constant volume fraction p and the constant interparticle distance g_f_, the geometrical dimensions of the representative cell (i.e., b and h) are uniquely determined.

For the selected RVE (Figure 4) and the selected boundary conditions in the form of Equation (8) (the unidirectional external field), the average intensity and flux of the field are defined by:

(10) $$\langle E_{\alpha }^{}\rangle =\sum_{m=1}^{TN}E_{\alpha m},\;\langle D_{\alpha }^{}\rangle =\sum_{m=1}^{TN}D_{\alpha m}$$

where TN denotes the total number of elements in the FEM mesh. Using the above relations, it is possible to compute four components of the average intensity and flux of the field for two types of boundary conditions demonstrated in Figure 4 (the 2D problem) or nine for the 3D analysis. We do not know in advance how many nonzero components in the property matrix $C_{\alpha \beta }^{1}$ occur. Therefore, for the linear problem, all components of the property matrix $C_{\alpha \beta }^{1}$ (nine for the 3D problem) can be derived directly from basic relationships presented in Table 1 where the appropriate components of the vectors **E** and **D** are replaced by their average values evaluated in the local cell (RVE).

For the non-linear problem (understood in the sense of the non-linear relation, Table 1), the components of the property matrix are computed by the comparison of the magnetic energy of the homogenized and of the original (Figure 4) unit cell combined with the Newton–Raphson method. The energy is represented as follows:

(11) $$U=\int_{\Omega_{RVE}}\int_{D}EdD\;d\Omega$$

However, it is necessary to know in advance the **D**–**E** characteristic curve for the inclusions. Commonly, the inclusion is modeled as isotropic material but for the unit cell the local property matrix can possess anisotropic properties as it is shown for example in Section 2.

**Figure 5:**
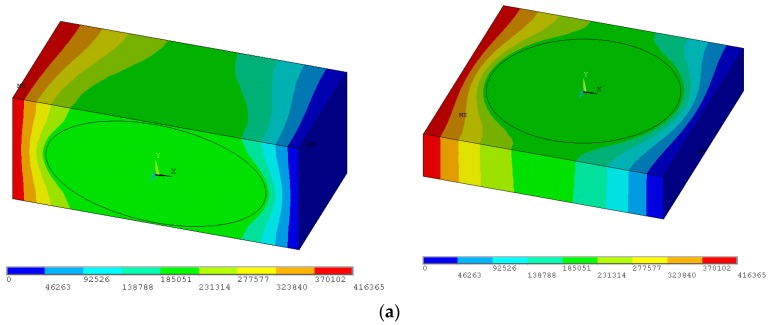

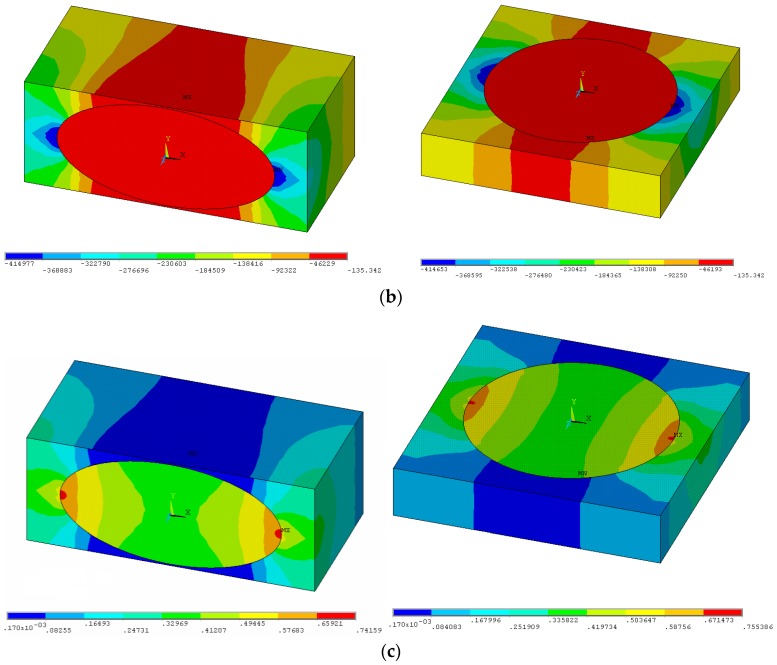
The local distributions of magnetic fields for the boundary conditions in Equation (8)—spheroids a_x_/a_y_ = 2, p = 0.3, r_p_/h = 0.485, c_p_ = 2000, c_0_ = 1: (**a**) distribution of the magnetic scalar potential ϕ; (**b**) distribution of the magnetic field intensity; and (**c**) distribution of the magnetic flux density.

Figure 5 represents the example the FEM analysis conducted for two-phase ferromagnetic composites made of phases having magnetic properties—the magnetostatic problem. The plots demonstrate the distribution of the magnetic flux density, the magnetic field and the potential ϕ as the external magnetic field is applied at the x direction—the boundary conditions in Equation (8). As can be seen, the variations of the potential inside the local cell (Figure 5a) result in non-homogeneous distributions of magnetic fields in all directions. The average values are evaluated by adding the values of each finite elements in the elementary cell.

**Figure 6:**
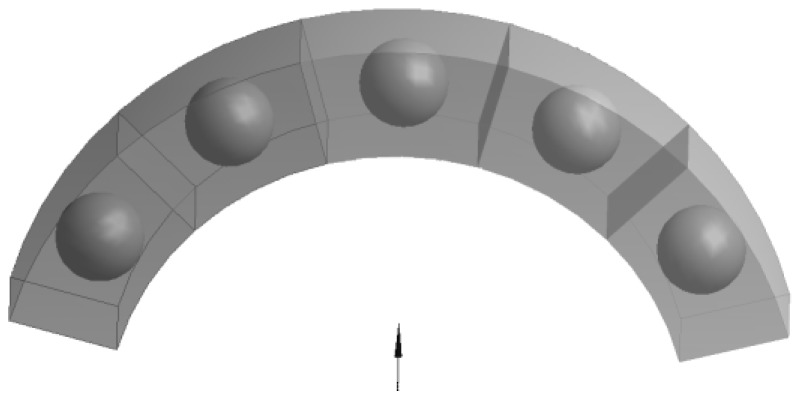
The system of 5 ferromagnetic particles—the external field is applied at the x, y or z direction.

For two-phase composites, the effective property tensor has analogous properties to, e.g., the tension modulus in elasticity: if the behavior is identical in three perpendicular directions, then it is isotropic. This conclusion points out a limitation of the use of constant second order tensors for the description of behavior. Indeed, e.g., for magnetism, many experimental observations reveal that cubic single crystals are not magnetically isotropic (see for instance Webster [30] for iron and nickel or Wang et al. [31] for Terfenol-D). In fact, in the experiments, the chains of particles do not have to be aligned in the direction of the external field, namely E_y_. We have some more compact aggregates. The microscopic analysis on the structure of the two-phase composites revealed that there were aggregates forming rather than chains of spheres that can be approximated by ellipsoids, stripes or cylinders. Therefore, it is interesting to verify the correctness of the introduced FE model in cases when the external field is rotated with respect to coordinates defining RVE in order to consider the relationship between the orientation of inclusions and to explore symmetries in the constitutive relations in Equation (1) and their relevance to the homogenized composite medium. In the case of a generalized anisotropic structure for which principal axis of external fields and the unit cell do not coincide, the property tensor should satisfy the classical transformation rules of the second rank tensors. To interpret and investigate those effects, let us analyze the form of the property (permeability) matrix for the 3D ring structure shown in Figure 6. The geometry of unit cells is defined in the cylindrical coordinate system but the external field is directed along the line joining the center of the central spherical inclusion and the center of the ring curvature.

**Figure 7:**
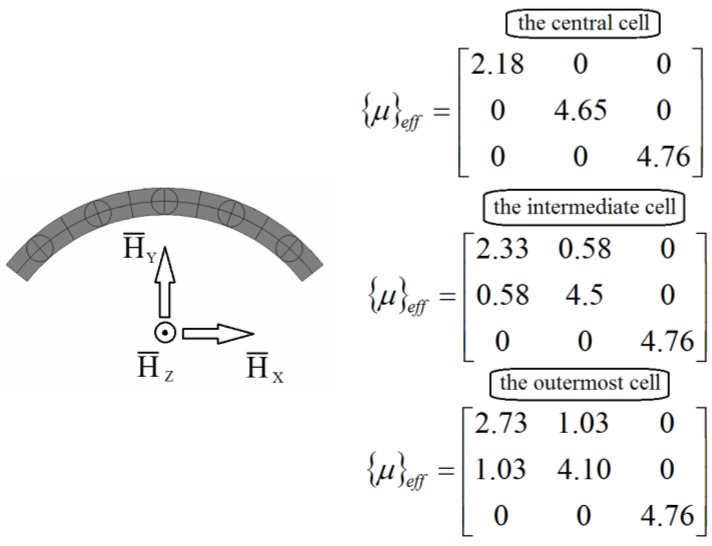
Boundary conditions and the local (transformed off-axis) values of the permeability matrix.

The assumed form of the system of the unit cells reflects the situation as the external field is not always parallel to the cell edges. It may occur for instance for clusters of inclusion. Figure 7 shows the assumed boundary conditions and the values of the permeability matrix terms (the magnetostatic problem). The terms c_αβ_ (α ≠ β) are not equal to zero, which means that it may be for instance the origin of clusters and of the inclusion aggregation at the beginning of magnetization. On the other hand, it may be easily verified that the terms of the property matrix satisfy the classical transformation rule:

(12) $${\langle C\rangle }^{Transf}=M\langle C\rangle M^{T},\;\mathrm{where}\;M=\left[\begin{array}{ccc} \cos\theta & \sin\theta & 0\\ -\sin\theta & \cos\theta & 0\\ 0 & 0 & 1 \end{array}\right]$$

and θ denotes the angle of rotation which is equal to 14.11^0^.

## 4. Numerical Results

The homogenization method is applied to various 2D and 3D test problems to evaluate the distributions of the terms of the property matrix and to compare theoretical predictions with numerical ones that take into account the finite dimensions of the unit cell. In the test problems, a sample two-phase composite material is composed of an isotropic matrix and the inclusions have the following material properties: c_p_ = 2000 and c_f_ = 1. The analysis is conducted for 2D and 3D unit cells to test the capability and limitations of the proposed model. Similar to the previous case, the numerical model corresponds to the analysis of MR fluids.

### 4.1. 2D Problems

**Figure 8:**
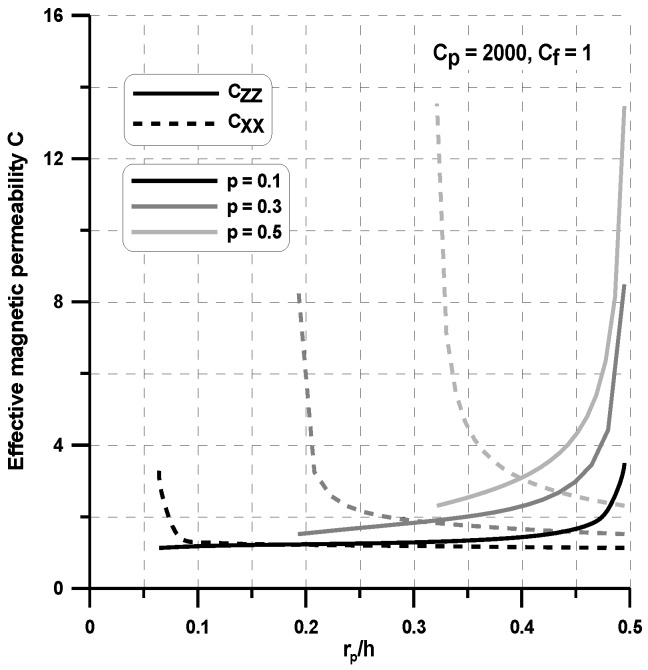
Variations of the effective permeability at two perpendicular directions with particle radius.

Let us consider a single circular particle surrounded by a nonmagnetic carrier fluid—the planar problem in Figure 4. This is a typical homogenization problem analyzed for the MR fluids (see, e.g., Simon et al. [32] for MR fluids). However, on the contrary to the cited work, we compute the four (the planar problem) permeability matrix coefficients. The off-axis terms are equal to zero, and two others are plotted in Figure 8 for various volume fractions.

**Figure 9:**
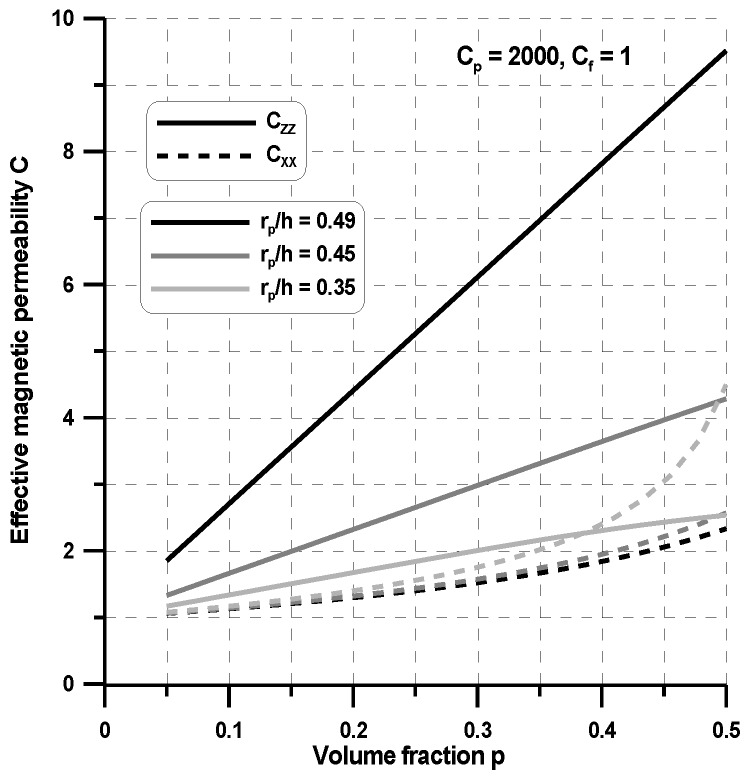
Variations of the effective permeability at two perpendicular directions with volume fractions.

As can be seen, the effective permeability c_zz_ decreases as the interparticle distance increases and it is the highest for the highest volume fraction p = 0.5. The decrease of the effective permeability c_xx_ is associated with the increase of the effective permeability c_zz_. Let us note that, for the constant volume fraction p, the variations of the ratio r_p_/h (or g_f_/r_p_) results in the change of the ratio b/r_p_ (see Equation (9)). Using the single unit cell (Figure 4), one can observe that, for the external magnetic field having the non-zero component H_z_ only, the chains of ferromagnetic particles are completely isolated since there is no interaction at the x direction. In fact, the experiments evidently demonstrate evidently that they form clusters of different shapes (see Bossis et al. [33]). In Figure 9, a relationship between the volume fraction and the effective permeability for arbitrary assumed value of ratio r_p_/h is depicted. As can be observed in the case of c_zz_, these relations can be considered as almost linear. In the case of c_xx_ for small values of volume fraction, they are also linear. However, for the values of volume fraction greater then p > 0.35, the effective permeability values increases exponentially. For both parameters, c_zz_ and c_xx_, the values are achieved for volume fraction p = 0.5.

### 4.2. 3D Problems

**Figure 10:**
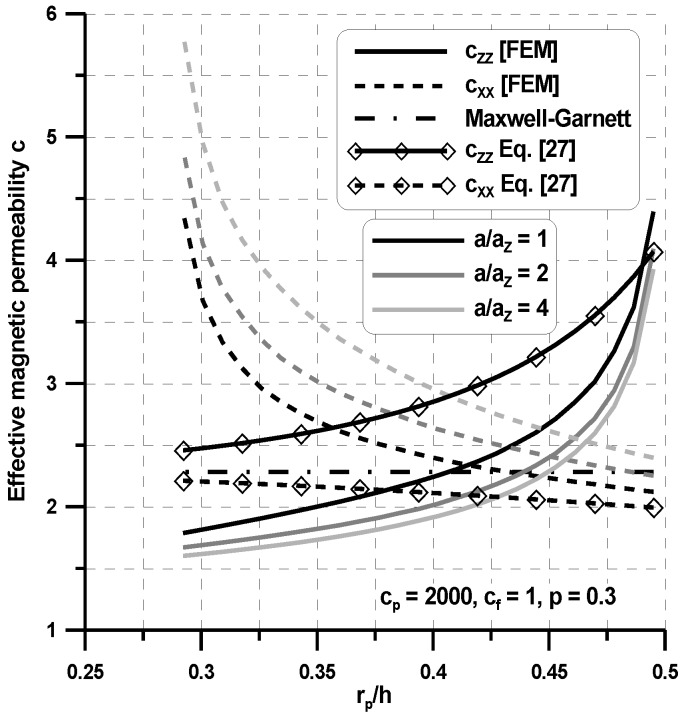
Variations of the permeability coefficients with interparticle vertical distance r_p_/h (transversely-isotropic body).

**Figure 11:**
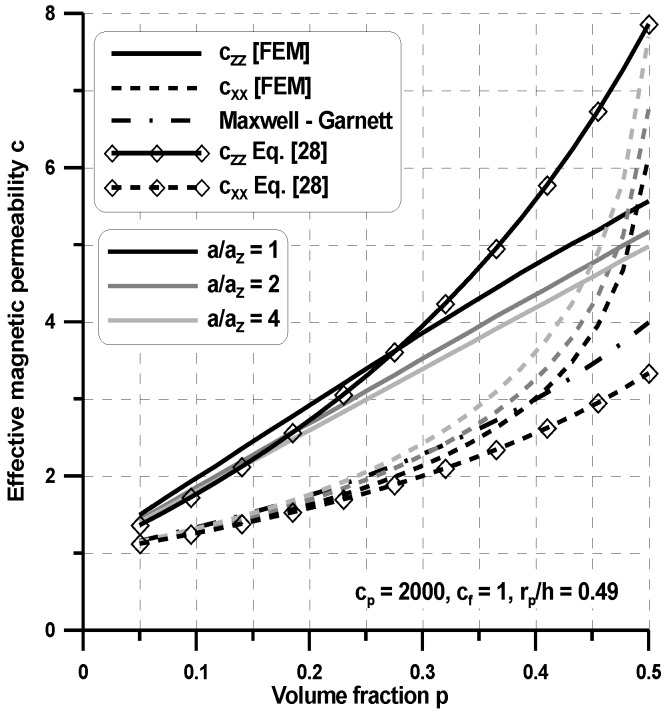
Variations of the permeability coefficients with volume fraction p (transversely-isotropic body).

Now, the 3D Laplace relationships shown in Table 1 have been solved for a 3D unit cell using the FEM package. The results for the variations of the property tensor components are demonstrated in Figure 10 and Figure 11 considering the assumption in Equation (9), which leads directly to the transversely isotropic properties of composites (i.e., c_xx_ = c_yy_). The plots are drawn both for spherical (a = a_x_ = a_y_ = a_z_) and spheroid (a = a_x_ = a_y_ ≠ a_z_) inclusions. For spherical inclusions, the distributions of the homogenized properties for the 3D case are similar to those for the 2D case (see Figure 8 and Figure 9). However, assuming the identical geometric ratios of the RVE, for the 3D case, the averaged values in the x direction are higher than those evaluated for the planar case, whereas the values in the z direction are almost identical. The shape of inclusions has a significant influence on the averaged values. For the increasing a_z_/h parameter, the effective property c_zz_ decreases, and c_xx_ increases since the x axis corresponds to the longer axis of spheroids. Figure 10 and Figure 11 show that the proposed model provides a transversely isotropic effective permeability, whereas the Maxwell–Garnett model gives an isotropic one. The Maxwell–Garnett model always yields the same estimates for any r_p_/h because it is insensitive to the microstructure. Thus, the Maxwell–Garnett model cannot be used for these composites because, even when the overall volume fraction is very small, the distance between particles of the same chain is so small that particle interactions cannot be disregarded. For the low volume fractions, the Maxwell–Garnett gives very good estimations in the x directions only (Figure 11). For spherical inclusions, as can be observed in Figure 10 and Figure 11, the effective field model gives much better approximations of the effective values evaluated with the use of the FE model than the Maxwell–Garnett model. However, the effectiveness of the effective field model decreases for the high particle interactions (a_z_/h < 0.1) and for the high volume fractions (p > 0.25). It is obvious that theoretical estimations, i.e., with the use of the Maxwell–Garnett model and the effective field model have limited applications in comparison with the FEM model since the first corresponds to the random (quasi-isotropic) structure of reinforced particles, and the second to the chain-like structure, i.e., in two directions the dimensions of the RVE tend to infinity.

## 5. Optimal Design

In the theoretical and numerical analysis, it is commonly assumed that the reinforcement particle has an ideal spherical form. However, as demonstrated in Figure 10 and Figure 11, the shape of the particle can significantly affect the effective material properties. Therefore, the development of optimized multifunctional composite materials becomes of great interest from the technological and theoretical viewpoints to all engineering fields. In general, such an approach belongs to the class of so-called shape optimization problems (see Muc et al. [34]). The broader description of optimization algorithms that can be applied herein is discussed by Muc [35].

This section designs such materials computationally using the method of parametric optimization. In particular, two-dimensional periodic two-phase composite materials are optimized for the optimal effective properties. To analyze the effects of different shapes, for simplicity, it is assumed that the particle is modeled as a superellipse (Figure 4) having the following form:

(13) $${\left(\frac{x}{a}\right)}^{n}+{\left(\frac{z}{b}\right)}^{n}=1$$

where a and b denote the superellipse semi-axis, and *n* is a parameter greater than 1. Having the constant volume fraction in the RVE and varying the n value, one can observe the change of the terms in the effective property matrix.

**Figure 12:**
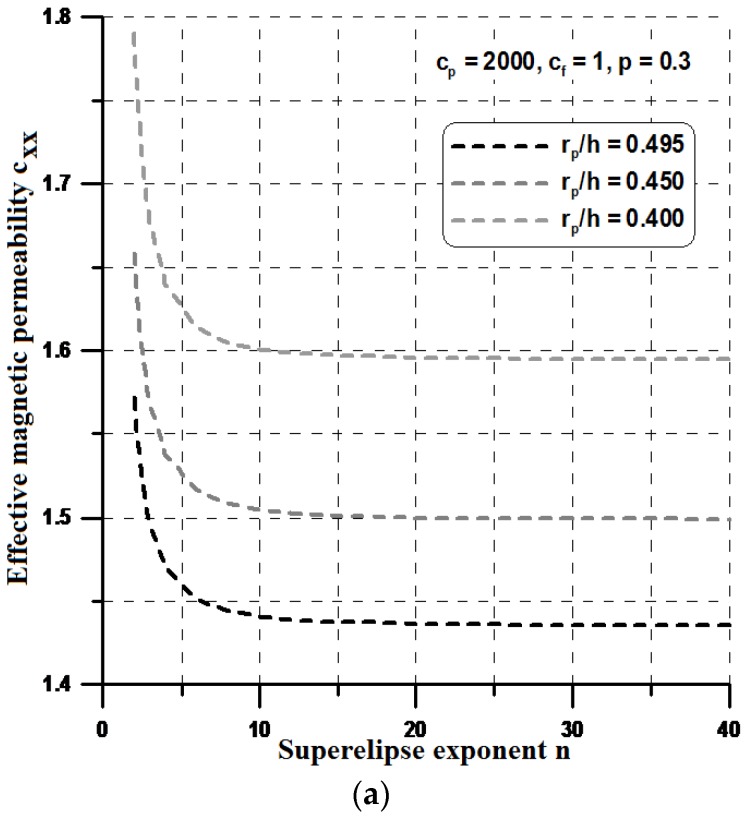

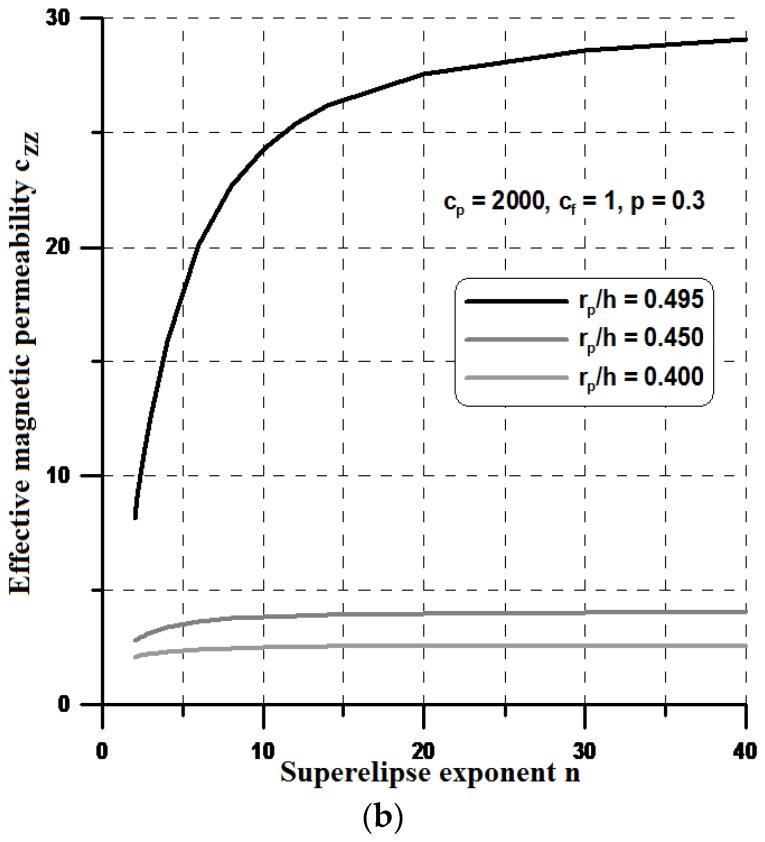
Variations of the effective properties with the particle shape—the constant volume fraction p: (**a**) the x direction; and (**b**) the y direction.

**Figure 13:**
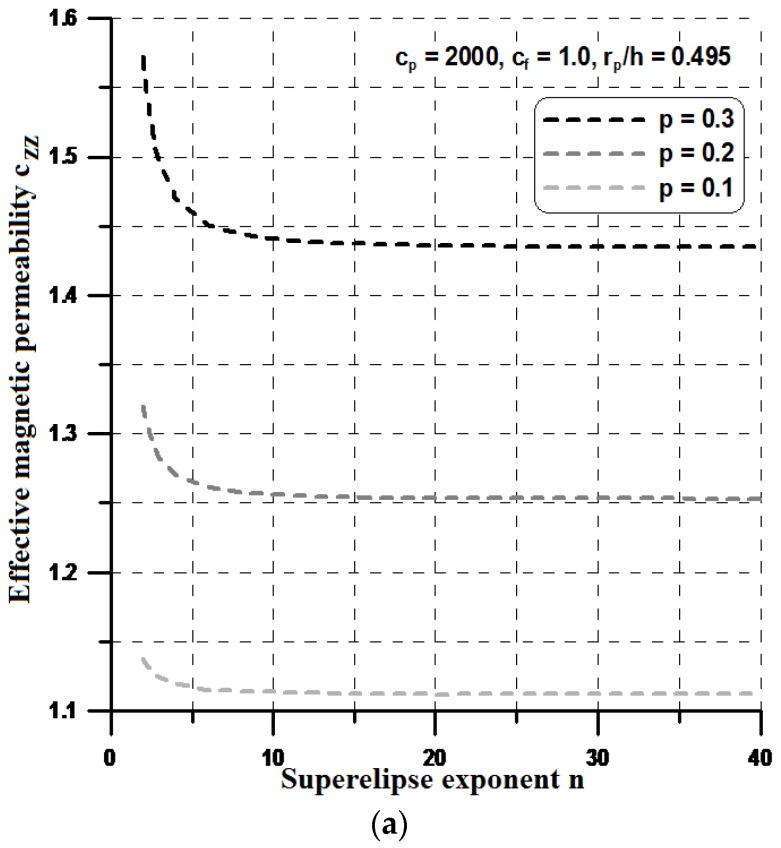

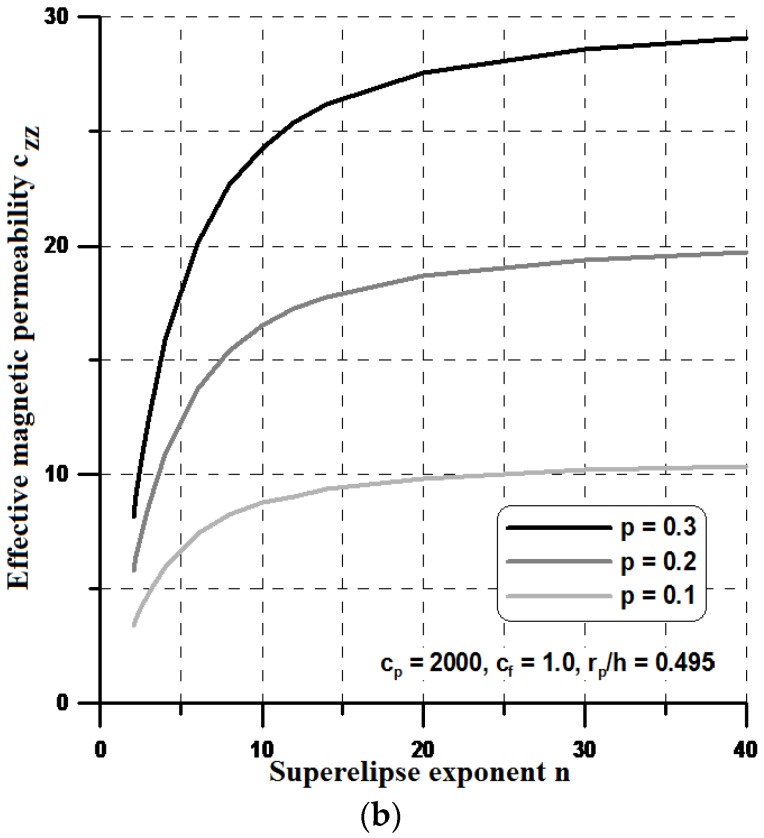
Variations of the effective properties with the particle shape—the constant interparticle distance r_p_/h: (**a**) the x direction; and (**b**) the y direction.

For higher values of the n parameter (n > 10), the shape resembles a rectangle and, in this case, both components of the effective permeabilities, i.e., c_yy_ and c_xx_ reach their optimal values (see Figure 12 and Figure 13). However, the optimal values of the effective properties are strongly dependent on the values of the geometrical ratios r_p_/h and the volume fractions p. Let us note that the maximal value of the term c_zz_ is much higher than those plotted previously in Section 4, and the values of c_zz_ are much lower. Therefore, it seems to be reasonable to conclude that the optimal rectangular form of the particles can prevent their aggregation in ellipsoids or cylinders instead of linear chains and in this sense, the theoretical effective field model may be applicable in the estimations of the effective properties. It is worth mentioning that the obtained optimal designs completely resemble those obtained by Guest and Prevost [36] for fluid transportation problem (the Darcy law). They concluded that the Schwartz P minimal surface is believed to be the maximum permeability structure in the 3D case. However, the authors of the cited paper assumed in advance the isotropic properties of the permeability matrix.

## 6. Concluding Remarks

For two-phase particulate composites, the compact, unique formulations and methods of solutions (theoretical and numerical) are presented in the paper. Although numerical examples deal with the analysis of magnetic phenomena, the methods and results can be easily extended to various other classes of problems.

The properties of a heterogeneous medium (two-phase composites) made of inclusions distributed in a locally periodic way in a matrix have been derived and studied. A uniform test external field is applied on the boundary of the composite, and then the averaged fields of the particles and matrix are derived theoretically by the Green’s function technique and then compared with FE results based on the numerical homogenization technique. An anisotropic effective property tensor is further provided. The effective property tensor of the composite medium is symmetric, positive definite, generally anisotropic, and depends on the microstructure both for 2D and 3D cases.

The results of the theoretical and computational (FEM) approximations are strongly dependent on the construction of the computational (representative) cell using specified dimensions and distributions of the reinforced particles, i.e., their location with respect to the centers of representative volume elements. The proposed methodology can be enriched by adding the interfacial elements between particles and matrices.

The proposed method can be successfully applied to the analysis of the non-linear problems, considering the non-linearity of the characteristic curves (e.g., **B**–**H**). From these models, it is found that the averaged property tensor components are strongly dependent on the dimensionless interparticle distance and the volume fraction.

In addition, this paper proposes a shape optimization methodology for designing multifunctional two-phase composite materials optimized for tensor property components. For the 2D problems, the optimal shape resembles rectangle with rounded edges. It is verified in this study that the optimal design based on the finite element analysis is a valid method for the output improvement of constructions.

The validity of the model should be also estimated by the inclusion of the uncertainties dealing with the shape of reinforcements or representative cells, e.g., using fuzzy sets [37].

It is important to emphasize that the underlying methodology of homogenization and optimization is quite general and can be applied to the design of arbitrary composite materials in different scales, i.e., micro-, meso- and even nanoscales.

The present analysis is conducted for single fields separately. In the global formulation of homogenization problems for the composite considered, the next step can be achieved by the compact formulation for coupled fields, i.e., the thermal and magneto-electric effects will be analyzed simultaneously.

## Figures and Tables

**Table 1:** The fundamental quantities and relationships for two-phase composites.

	Magnetostatics	Electrostatics	Heat Flow	Diffusion	Porous Media
	Definition of scalar potentials ϕ				
ϕ	Scalar potential ϕ (A)	Electric potential V (Volt)	Temperature T (°K)	Substance concentration γ (mol/m^3^)	Hydrostatic pressure p (Pa)
	Definition of vector fields in the vacuum $E_{\alpha }=\nabla_{\alpha }\varphi$, $rot_{\alpha \beta }E_{\beta }=0$				
E_α_	Intensity of magnetic field H_α_ (A/m)	Intensity of electric field E_α_ (V/m)	Not used	Not used	Not used
	Definition of material properties C_αrβ_				
C_αβ_	Magnetic permeability coefficients μ_0_μ_αβ_ (Tm/A)	Electric permittivity coefficients ε_0_ε_αβ_ [(A s)/(V m)]	Heat conduction coefficients k_αβ_ [W/(°K m)]	Diffusion coefficients D_αβ_ (m^2^/s)	Proportionality coefficients K_αβ_/η [m^2^/(Pa s)] *
	Definitions of vector fields in a particular medium D_α_				
D_α_	Magnetic flux density B_α_ (T)	Electric flux D_α_ (C/m^2^)	Not used	Not used	Not used
	Constative relations $D_{\alpha }=C_{\alpha \beta }E_{\beta }$				
	Physical relations $\nabla_{\alpha }D_{\alpha }=0$				
	Gauss’s Law for magnetism $\nabla_{\alpha }\left(\mu_{0}\mu_{\alpha \beta }H_{\beta }\right)=0$	Gauss’s Law $\nabla_{\alpha }\left(\varepsilon_{0}\varepsilon_{\alpha \beta }E_{\beta }\right)=\rho$	Fourier’s Law $\nabla_{\alpha }\left(k_{\alpha \beta }\nabla_{\beta }T\right)+Q=0$	Fick’s Law $\nabla_{\alpha }\left(D_{\alpha \beta }\nabla_{\beta }\gamma \right)=0$	Darcy’s Law $\nabla_{\alpha }\left[\left(K_{\alpha \beta }/\eta \right)\nabla_{\beta }p\right]=0$

μ_0_, magnetic permeability of vacuum, μ_0_ = 4π × 10^−7^ (Tm/A); ε_0_, electric permittivity of vacuum, ε_0_ = 8.854187817 × 10^−12^ [(A s)/(V m)]; * η, dynamic viscosity of fluid (Pas).

## Appendix A. Mathematical Formulation and Derivation of the Effective (Homogenized) Material Properties

Let us consider a homogeneous medium with properties described by a tensor $C_{\alpha \beta }^{0}$. The medium contains the region $\Omega^{\left(1\right)}$ (inhomogeneity) with another property tensor $C_{\alpha \beta }^{}$. The intensity E(x) and the flux D(x) of the field in the medium are described by the following system of differential equations:

(A1) $$\nabla_{\alpha }D_{\alpha }\left(x\right)=-q(x),\;D_{\alpha }\left(x\right)=C_{\alpha \beta }(x)E_{\beta }(x),\;{\mathrm{rot}}_{\alpha \beta }E_{\beta }(x)=0$$

where q(x) is a scalar density of the field sources. The third equation is satisfied automatically if the field E_α_(x) is the gradient of a scalar function φ(x) called the potential of the field:

(A2) $$E_{\alpha }\left(x\right)=\nabla_{\alpha }\varphi \left(x\right)$$

The physical meaning of the potential φ(x) depends on the problem under considerations. It means the temperature field for heat transfer problems, the potential of the electric (magnetic) field for the electrostatics (magnetostatics, respectively) fields.

The tensor $C_{\alpha \beta }^{}$ represents the local properties of the inclusion (Figure 1b) and is defined as the sum:

(A3) $$C_{\alpha \beta }(x)=C_{\alpha \beta }^{0}+C_{\alpha \beta }^{1}(x)$$

Thus, if we consider a homogeneous medium with the property tensor C^0^ containing a set of inclusions with the property tensor C, the system of differential Equations (A1) and (A2) may be reduced to integral equations for the fields E(x) inside the inclusions, i.e.,

(A4) $$E_{\alpha }(x)+\int_{\Omega_{\left(k\right)}^{1}}K_{\alpha \beta }(x-x^{\prime })C_{\beta \mu }^{1}(x^{\prime })E_{\mu }(x^{\prime })V(x^{\prime }){\mathrm{dx}}^{\prime }=E_{\alpha }^{0}\left(x\right)$$

where V(x′) is a characteristic function for the *k*th inclusion that is equal to one if the variable x′ belongs to the region $\Omega_{\left(k\right)}^{1}$ occupied by the *k*th inclusion and to zero if x′ does not belong to the domain $\Omega_{\left(k\right)}^{1}$ (see Figure 1). Here, E_0_ is the external field in the medium without the inclusion (C^1^(x) = 0) by the action of the same sources of the field. The kernel K(x) is determined in the classical manner:

(A5) $$K(x)=-\nabla_{\alpha }\nabla_{\beta }\;G(x)$$

where G(x) is the Green function for the infinite homogeneous medium with the property tensor C^0^. The Green function satisfies the equation:

(A6) $$\nabla_{\alpha }C_{\alpha \beta }^{0}\nabla_{\beta }\;G(x)=-\delta (x)$$

The equivalence between the relations in Equations (A4) and (A1)–(A3) can be proven under two additional assumptions:

- With the use of the potential φ(x), the solution of Equation (A1) can be decomposed as follows: $\varphi \left(x\right)=\varphi^{0}\left(x\right)+\varphi^{1}\left(x\right)$, where $\varphi^{0}\left(x\right)$ is the potential in the medium without the inclusion, and $\varphi^{1}\left(x\right)$ is the perturbation of the potential due to the presence of the inclusion that tends to zero when |x|→∞.
- The potential $\varphi^{0}\left(x\right)$ satisfies the relation analogous to Equation (A1), i.e., $\nabla_{\alpha }C_{\alpha \beta }^{0}\nabla_{\beta }\;\varphi^{0}(x)$ = −q(x).

The solution of this equation for an arbitrary anisotropic medium takes the following form:

(A7) $$G(x)=\frac{1}{4\;\pi \;r(x)},\;r(x)=\sqrt{\det C^{0}x_{\alpha }B_{\alpha \beta }^{0}x_{\beta }},\;B^{0}={\left(C^{0}\right)}^{-1}$$

The simplest version of the effective field method is based on the hypothesis that the local external field $E_{\beta }^{*}(x)$, which acts on each inclusion, is the same for all inclusions, i.e., the field $E_{\alpha }(x)$ inside each inclusion can be presented in the following way:

(A8) $$E_{\alpha }(x)=\Lambda_{\alpha \beta }^{E}\left(x\right)E_{\beta }^{*}(x)$$

where the tensor $\Lambda_{\alpha \beta }^{E}\left(x\right)$ is determined from the solution of the problem for isolated inclusion in the medium with the property tensor C^0^ by the action of the field $E_{\beta }^{*}(x)$, and, for ellipsoidal inclusions, it takes the following form:

(A9) $$\Lambda_{\alpha \beta }^{E}\left(x\right)={\left(\delta_{\alpha \beta }+A_{\alpha \lambda }^{0}C_{\lambda \beta }^{1}\right)}^{-1}$$

where

(A10) $$A_{\alpha \beta }^{0}=A_{1}^{0}e_{\alpha }^{1}e_{\beta }^{1}+A_{2}^{0}e_{\alpha }^{2}e_{\beta }^{2}+A_{3}^{0}e_{\alpha }^{3}e_{\beta }^{3},\;A_{n}^{0}=\frac{a_{1}a_{2}a_{3}}{2c_{0}}\int_{0}^{\infty }\frac{d\sigma }{\left(a_{n}^{2}+\sigma \right)\sqrt{\left(a_{1}^{2}+\sigma \right)\left(a_{2}^{2}+\sigma \right)\left(a_{3}^{2}+\sigma \right)}},\;n=1,2,3$$

a_1_, a_2_, and a_3_ are the semiaxes of an ellipsoid, and $e_{\alpha }^{n}$ are the unit vectors of the ellipsoid principal axes, the orientation of which is given by the normal m. For a spheroid inclusion with the semiaxes a_1_ = a_2_ = a, a_3_, the tensor A^0^ takes the following form:

(A11) $$A_{\alpha \beta }^{0}=A_{1}^{0}\theta_{\alpha \beta }+A_{3}^{0}m_{\alpha }m_{\beta },\;\theta_{\alpha \beta }=\delta_{\alpha \beta }-m_{\alpha }m_{\beta },\;A_{1}^{0}=\frac{1}{c_{0}}f_{0}\left(\gamma \right),\;A_{3}^{0}=\frac{1}{c_{0}}\left(1-2f_{0}\left(\gamma \right)\right),\;\gamma =\frac{a}{a_{3}}>1,\;f_{0}\left(\gamma \right)=\frac{1-g}{2\left(1-\gamma^{2}\right)},\;g=\frac{\gamma^{2}}{\sqrt{\gamma^{2}-1}}\;\mathrm{arctg}\left(\sqrt{\gamma^{2}-1}\right)$$

The effective local exciting field $E_{\beta }^{*}(x)$ acting on the *k*th inclusion is the sum of the external field $E_{\beta }^{0}(x)$ applied to the medium and the field induced by others surrounding inclusions. The field $E_{\beta }^{}(x)$ inside the isolated *k*th inclusion being in the background matrix and caused by the action of the field $E_{\beta }^{*}(x)$ is defined by Equation (A4) where $E_{\beta }^{0}(x)$ is replaced by $E_{\beta }^{*}(x)$. The field induced in the region $\Omega_{\left(k\right)}^{1}$ of the *k*th inclusion by all surrounding inclusions can also be represented using Equation (A4) in the following form:

(A12) $$E_{\alpha }^{*}\left(x\right)=E_{\alpha }^{0}\left(x\right)-\int_{\Omega_{\left(i\right)}^{1}}K_{\alpha \beta }(x-x^{\prime })C_{\beta \mu }^{1}(x^{\prime })\Lambda_{\mu \lambda }^{E}\left(x\right)V(x;x^{\prime })E_{\lambda }^{*}(x^{\prime }){\mathrm{dx}}^{\prime }$$

(A13) $$V\left(x;x^{\prime }\right)=\sum_{i\neq k}V_{i}(x^{\prime })\;\mathrm{when}\;x\in \Omega_{\left(k\right)}^{1}$$

Averaging Equation (A12), assuming that the field $E_{\alpha }^{0}\left(x\right)$ is fixed in the problem, i.e.,

(A14) $$\langle E_{\alpha }^{0}\left(x\right)\rangle =E_{\alpha }^{0}$$

and identifying the conditional mean $\langle E_{\alpha }^{*}\left(x\right)|x\rangle$ with the effective field $E_{\alpha }^{*}$, we finally obtain the following relation:

(A15) $$E_{\alpha }^{*}=E_{\alpha }^{0}-p\int K_{\alpha \beta }(x-x^{\prime })P_{\mu \lambda }^{0}\Psi (x-x^{\prime })E_{\lambda }^{*}{\mathrm{dx}}^{\prime }\;\mathrm{where}\;P_{\beta \lambda }^{0}=\frac{1}{\langle V_{i}\rangle }\langle \int_{\Omega_{\left(i\right)}^{1}}C_{\beta \mu }^{1}(x)\Lambda_{\mu \lambda }^{E}\left(x\right)\mathrm{dx}\rangle$$

The symbol 〈.〉 denotes the averaging, and the function Ψ is defined as follows:

(A16) $$\Psi \left(x,x^{\prime }\right)=\frac{\langle V(x,x^{\prime })|x\rangle }{\langle V(x)\rangle }\;\mathrm{where}\;\langle f(x)|x\rangle =\frac{\langle f(x)V(x)\rangle }{\langle V(x)\rangle }$$

p is the volume concentration of inclusions and it is equal to 〈V(x)〉. The symbol 〈⋅|x〉 denotes the averaging over the realizations of the random set of inclusions by the condition x∈V. The solution of Equation (A15) can be expressed in the following way:

(A17) $$E_{\alpha }^{*}=E_{\alpha }^{0}{\left(\delta_{\alpha \beta }+{\mathrm{pK}}_{\alpha \lambda }^{\Psi }P_{\lambda \beta }^{0}\right)}^{-1},\;K_{\alpha \lambda }^{\Psi }=\int_{\Omega_{\left(i\right)}^{1}}K_{\alpha \beta }(x-x^{\prime })\Psi (x-x^{\prime }){\mathrm{dx}}^{\prime }$$

Multiplying Equation (A4) by the property tensor C^0^ and using the definition in Equation (A1) of the flux tensor D(x) (for the inclusion and the medium) after a set of transformation of the result, it is possible to derive the average of the flux tensor, i.e.,

(A18) $$\langle D_{\alpha }^{}\left(x\right)\rangle =\left[C_{\alpha \beta }^{0}+P_{\alpha \lambda }^{0}{\left(\delta_{\lambda \beta }+{\mathrm{pK}}_{\lambda \mu }^{\Psi }P_{\mu \beta }^{0}\right)}^{-1}\right]E_{\alpha }^{0}$$

On the other hand, using Equations (A1) and (A3), the tensor of the effective (homogenized) properties of the composite may be defined as follows:

(A19) $$\langle D_{\alpha }(x)\rangle =C_{\alpha \beta }^{*}\langle E_{\beta }(x)\rangle ,\;C_{\alpha \beta }^{*}=\langle C_{\alpha \beta }\left(x\right)\rangle$$

Combining Equations (A18), (A19) and (A14), one can find that the tensor of the effective properties of two-phase composite is represented by the relation:

(A20) $$C_{\alpha \beta }^{*}=\langle C_{\alpha \beta }\left(x\right)\rangle =\left[C_{\alpha \beta }^{0}+{\mathrm{pP}}_{\alpha \lambda }^{0}{\left(\delta_{\lambda \beta }+{\mathrm{pK}}_{\lambda \mu }^{\Psi }P_{\mu \beta }^{0}\right)}^{-1}\right]$$

Taking into account Equations (A12) and (A15) as well as assuming that the tensor $C_{\beta \mu }^{1}$ is independent on the x variable, the above relation takes the following form:

(A21) $$C_{\alpha \beta }^{*}=\langle C_{\alpha \beta }\left(x\right)\rangle =\left[C_{\alpha \beta }^{0}+{\mathrm{pC}}_{\alpha \beta }^{1}{\left\{\delta_{\lambda \beta }+\left(A_{\lambda \mu }^{0}+{\mathrm{pK}}_{\lambda \mu }^{\Psi }\right)C_{\mu \beta }^{1}\right\}}^{-1}\right]$$

If the ratio γ has the same order as the inclusion aspect ratio, $K_{\lambda \mu }^{\Psi }=-A_{\lambda \mu }^{0}$ and the above equation is further reduced. Thus, as may be easily observed, the two-phase composite has isotropic, transversely-isotropic or anisotropic properties and they are directly dependent on the form of the operator $A_{\lambda \mu }^{0}$—under the assumption that both the matrix and the inclusion have isotropic properties.
